# Cerenkov luminescence imaging (CLI) for image-guided cancer surgery

**DOI:** 10.1007/s40336-016-0183-x

**Published:** 2016-05-24

**Authors:** M. R. Grootendorst, M. Cariati, A. Kothari, D. S. Tuch, A. Purushotham

**Affiliations:** 1 Department of Research Oncology, 3rd Floor Bermondsey Wing, King’s College London, London, SE1 9RT UK; 2 Department of Breast Surgery, 3rd Floor Tower Wing, Guy’s Hospital, London, SE1 9RT UK; 3 Lightpoint Medical Ltd, The Island, Moor Road, HP5 1NZ Chesham, UK

**Keywords:** Cerenkov luminescence imaging, Image-guided surgery, Cerenkov luminescence endoscopy, Tumour margins, Lymph nodes

## Abstract

Cerenkov luminescence imaging (CLI) is a novel molecular optical imaging technique based on the detection of optical Cerenkov photons emitted by positron emission tomography (PET) imaging agents. The ability to use clinically approved tumour-targeted tracers in combination with small-sized imaging equipment makes CLI a particularly interesting technique for image-guided cancer surgery. The past few years have witnessed a rapid increase in proof-of-concept preclinical studies in this field, and several clinical trials are currently underway. This article provides an overview of the basic principles of Cerenkov radiation and outlines the challenges of CLI-guided surgery for clinical use. The preclinical and clinical trial literature is examined including applications focussed on image-guided lymph node detection and Cerenkov luminescence endoscopy, and the ongoing clinical studies and technological developments are highlighted. By intraoperatively guiding the oncosurgeon towards more accurate and complete resections, CLI has the potential to transform current surgical practice, and improve oncological and cosmetic outcomes for patients.

## Introduction

### Cancer surgery

The International Agency for Research on Cancer (IARC) reports that 14.1 million new cancer cases were diagnosed in 2012 worldwide, with 8.2 million cancer-related deaths. By 2030, these figures will grow to 21.7 million new cases and 13 million deaths, simply due to population growth and ageing [2]. Of the estimated 21.7 million global new cancer patients in 2030, 17.3 million, or approximately 80 %, will need surgery as the main form of treatment [3].

For cancer surgery to have curative intent, complete tumour resection (i.e. excision of all cancer tissue with no residual loco-regional disease) is mandatory. To achieve this, surgeons try to identify a tumour’s extent, and aim to excise the lesion with a surrounding margin of healthy tissue. In an effort to minimise functional loss and/or cosmetic impairment, the goal is to remove the least possible amount of healthy tissue without compromising oncological safety [4].

Palpation and visual inspection—combined with a surgeon’s experience and judgement—are currently the only widely available ‘modalities’ to guide resection. These are frequently inaccurate at discriminating between malignant and normal tissue, resulting in positive tumour margin rates of up to 50 % in some cancers [5–7]. Positive margins are associated with a higher risk of local recurrence and poor prognosis [8–11]. Adjuvant treatments such as radiotherapy, hormone therapy or chemotherapy, and repeat operations to excise residual disease are often indicated to reduce the likelihood of local recurrence, but these treatments can impact on quality of life by causing significant physical and emotional distress, and suboptimal cosmetic outcome [12, 13].

With the above in mind, continuing efforts have been made to assist surgeons in the process of determining which tissue needs to be excised during cancer surgery. Currently used clinical techniques include ultrasonography, specimen radiography, and intraoperative histology and cytology techniques. Although all of these techniques are used to varying degrees in cancer surgery, none has quite solved the Goldilocks problem of margins, due to limitations in sensitivity, specificity, accuracy, or costs [14, 15].

#### Cerenkov luminescence imaging

Cerenkov luminescence imaging (CLI) is a novel imaging modality that has great potential for image-guided surgery in general, and the issue of surgical margins in particular. CLI is based on the detection of Cerenkov photons emitted by positron emission tomography (PET) imaging agents. Cerenkov photons are emitted by a charged particle (positron or electron) when travelling through a dielectric medium at a velocity greater than the velocity of light in that medium. The Cerenkov phenomenon seems to have been first observed by Marie Curie in the late 19th century. In her biography, she describes observing a pale blue glow from the radium-containing bottles in her laboratory. The first person to systematically describe Cerenkov radiation was Pavel Cerenkov, and together with Il’ja Mikhailovic Frank and Igor Yevgenyevich Tamm who developed the theoretical framework, they won the Nobel Prize in Physics in 1958 for their contribution to the discovery of the Cerenkov effect. In the lay mind, Cerenkov radiation is known as the blue glow in the cooling water basins that surround nuclear reactors.

By detecting the optical photons from PET imaging tracers, CLI combines optical and molecular imaging. Robertson et al. were the first who demonstrated that CLI with PET agents can be used to image cancer in vivo [16], and since then, this technology has rapidly emerged in the field of biomedical imaging. In recent years, several review papers have outlined the various applications of CLI including its use in Cerenkov luminescence imaging dosimetry (CLID), radionuclide therapy monitoring, tumour response monitoring and photoactivation therapy [17–21]. An in-depth explanation of the complex physics underlying Cerenkov radiation and CLI has also been reported [22, 23].

The aim of this review paper is to provide an overview on the use of CLI for image-guided interventions with a specific focus on image-guided cancer surgery. The first section of this paper outlines the characteristics of Cerenkov radiation and CLI. Rather than describing these characteristics using complex physical equations as already done by others, this review provides a simplified explanation with an emphasis on the features that are relevant to image-guided surgery. The second section of this paper contains an overview of the published work in this field to date, and the last section will highlight the ongoing clinical studies and technological developments.

## Cerenkov radiation: the basics

**Fig. 1:**
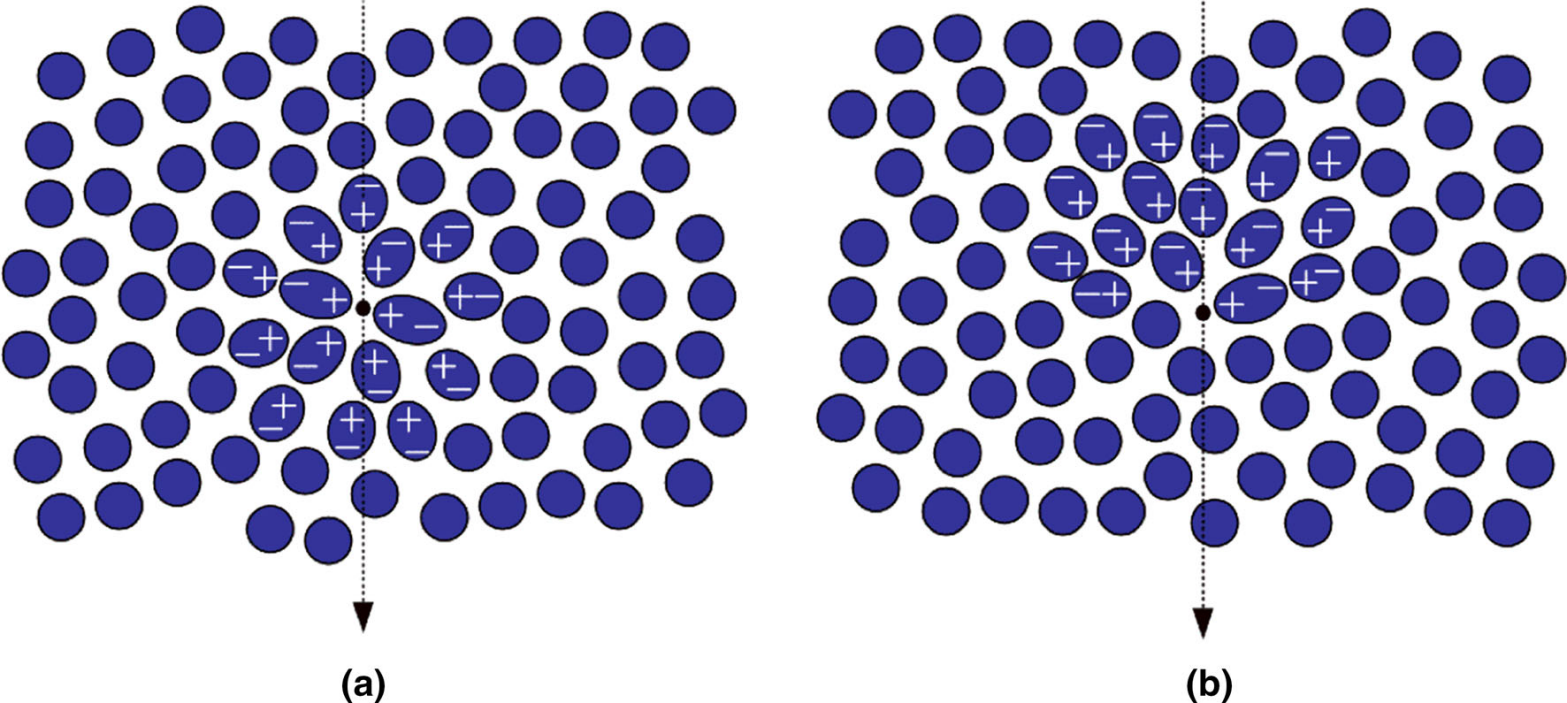
A charged particle, in this case an electron, passing through a dielectric medium with **a** a particle speed (*ν*) lower than speed of light in that medium (*c*/*η*), **b** a particle speed larger than speed of light in that medium. The condition such that Cerenkov luminescence is produced along the particle’s track requires *ν* ≥ *c*/*η*

Cerenkov radiation is produced when a charged particle travels through a dielectric medium, i.e. a medium that can be polarised by an electric field, with a speed faster than the speed of light in that medium [24]. When propagating, the charged particle (a positively charged positron or negatively charged electron) induces a local polarisation by displacing the positive and negative charges of the atoms in the medium (Fig. 1). In a situation where the particle’s velocity does not exceed the speed of light in that medium, the polarisation field surrounding the particle is perfectly symmetrical, and there is no electric field at larger distances. The net result is that no Cerenkov radiation is emitted. When the particle’s speed exceeds the speed of light, however, the polarisation becomes asymmetrical along the track of the particle, resulting in a dipole electric field at larger distances from the particle. As the particle passes the electrons of the atoms return to their ground state, thereby emitting the transferred energy as optical photons that are known as Cerenkov radiation. Thus, Cerenkov radiation is produced as secondary emission; it is not the charged particle generating light, but the medium as a reaction to the particle.

For Cerenkov radiation to be emitted, the charged particle needs to exceed a certain energy threshold. This threshold is expressed by ${\text{v }} \ge c/\eta$, where *ν* is the charged particle’s velocity, *c* is the speed of light in vacuum, and *η* is the refraction index of the medium. From this expression, it becomes clear that the Cerenkov threshold is related to the refractive index of the medium. Using the relationship between the velocity of the particle and its energy as described by equations 2 and 3 in Gill et al., it is found that in water with a refractive index of 1.33, the threshold is 0.264 MeV [25]. In soft tissues, the refractive index typically ranges from 1.36 to 1.40, resulting in a threshold for the production of Cerenkov radiation of approximately 0.219–0.250 MeV. These thresholds are lower than the beta particle energies from radionuclides used in PET, and these radionuclides thus emit Cerenkov radiation in both water and tissue [26]. As the charged particle travels through the medium, it loses energy due to interactive processes with its surroundings including absorption and scattering, and eventually, its energy falls below the threshold, and Cerenkov light is no longer produced. For the much heavier alpha particles, the Cerenkov threshold in water and tissue is 1926 and 1673 MeV, respectively [27]. Although none of the energies from existing alpha-emitting radionuclides come near this threshold (typical alpha particle energies range between 3 and 7 MeV), studies have shown emission of Cerenkov photons by alpha emitters [27–29]. There are two explanations for this observation, depending on the type of radionuclide: either photons arise from the short-lived beta-emitting daughter radionuclides of some alpha emitters (e.g. Actinium-225), or they are produced by electrons that arise from Compton scattered high-energy gamma photons. Regardless of the mechanism, Cerenkov radiation from alpha emitters is, thus, produced indirectly by secondary beta particles. The pure gamma-emitter Technetium-99 m (^99m^Tc) is also able to produce optical photons as shown by several groups [30–32]. Although the mechanism of this optical emission is not yet fully understood, it is assumed to be from OH radicals that are excited by the low energy Compton electrons [30] or from gamma excitation of the luminophores that are present in ^99m^Tc based tracers (e.g. the amino acids in ^99m^Tc-macroaggregates albumin) [32]. This form of luminescence is known as radioluminescence and differs from Cerenkov radiation; it has a different wavelength spectrum, and its signal intensity is lower in tissue [33]. The latter may provide additional challenges for its use in image-guided interventions. In the remainder of this review, our focus will, therefore, solely be on Cerenkov radiation.

The number of Cerenkov photons *N* emitted per distance travelled *x* can be calculated using equation 1, which is derived from the Frank–Tamm equation [25]:

$$\frac{{{\text{d}}N}}{{{\text{d}}x}} = 2\pi \alpha \left( {1 - \frac{1}{{\beta^{2} \eta^{2} }}} \right)\mathop \int \limits_{\lambda 1}^{\lambda 2} \frac{1}{{\lambda^{2} }}{\text{d}}\lambda$$

Here, *α* is the fine structure constant (1/137), *β* is the ratio between particle’s velocity and the speed of light in vacuum (ν/*c*), and the integral is over the interval *λ*_1_ to *λ*_2_. From this equation, it follows that the intensity of the Cerenkov radiation depends on a particle’s velocity, and thus, its energy. Fluorine-18 (^18^F), the most commonly used radionuclide in PET imaging, has an average and maximum β-energy of 250 and 633 keV, respectively. As a result, only 47 % of the decays produce a positron that exceeds the energy threshold for production of Cerenkov radiation in water [22]. Yttrium-90 (^90^Y), a radionuclide often used in radiation therapy, has a much higher average and maximum β-energy of 934 keV and 2.28 MeV, respectively, and 90 % of its produced electrons are above the Cerenkov threshold in water. Gill et al. recently studied 47 radionuclides widely used in nuclear medicine, and used Monte Carlo simulations to quantify the expected Cerenkov light yield (photons/decay) for each radionuclide in tissue (*η* = 1.4) [25]. They found that ^18^F emits 2.58 photons per decay in tissue; approximately 23 times less than the 58.5 photons per decay emitted by ^90^Y. The light yield from some commonly used radionuclides in order from high to low is shown in Table 1. Although it is important to realise that the reported light yields do not take into account the wavelength-dependent absorption and wavelength-dependent scattering that would occur in human tissue—this would reduce the number of detectable photons—it is clear that the signal intensity of CLI can be improved significantly using higher-energetic isotopes. However, even with the use of such isotopes, the Cerenkov light yield from a single radioactive decay process is low in comparison to, for example, the light yield from a single fluorescent molecule. Fluorescein and Indocyanine green (ICG), fluorophores used in fluorescence image-guided surgery, emit roughly three orders of magnitude more photons [34]. This low light yield requires strict control of the light environment to obtain a sufficient signal-to-background ratio (SBR) when using CLI in an intraoperative setting as explained below.

**Table 1:** Relevant characteristics of Cerenkov radiation and CLI for image-guided cancer surgery

Cerenkov radiation definition	Optical radiation emitted by charged particles when travelling through a dielectric medium with a speed larger than the speed of light in that medium
Threshold energy for Cerenkov radiation emission [25]	Water (*η* = 1.33): 0.264 MeV Biological tissue (*η* = 1.36–1.40): 0.219–0.250 MeV
Cerenkov radiation is emitted by	β^+^, β^−^, and α-emitting radionuclides
Cerenkov intensity from radionuclides most commonly used in clinic in order from high to low [25]	^90^Y > ^68^Ga > ^15^O > ^124^I > ^11^C > ^89^Zr > ^18^F > ^131^I > ^64^Cu
Cerenkov radiation spectrum [16]	350–900 nm
Fundamental resolution [22]	0.3–2.00 mm
Camera requirements for Cerenkov radiation detection	High-sensitivity optical cameras with single-photon detection capability
Typical penetration depth in tissue [70]	~1–2 cm
Typical CLI acquisition times	1–5 min
Types of images acquired with CLI	Photographic image: anatomical information Functional image: information on the uptake and location of the radiopharmaceutical
Advantages of CLI for image-guided cancer surgery	Ability to use clinically approved tumour-targeted radiopharmaceuticals Potential for multi-modality imaging with the same tracer: preoperative imaging with gamma-camera, PET or SPECT, intraoperative imaging using CLI ± beta-probe or gamma-probe Small form factor of CLI equipment allowing implementation of CLI technology in intraoperative specimen chamber, flexible endoscope and rigid laparoscope External excitation source not required: less tissue autofluorescence
Challenges of CLI for image-guided cancer surgery	Faint signal Light-tight imaging conditions required Radiation dose to patient and staff Strict regulations for use of radiotracers Complex logistics that requires close multi-disciplinary team work

Another characteristic of Cerenkov light is its broad emission spectrum that ranges from approximately 350 to 900 nm [16]. The light intensity is inversely proportional to the square of the wavelength (1/*λ*^2^). This is why Cerenkov radiation is strongest towards the blue end of the visible spectrum, and hence why Cerenkov radiation appears blue.

The fundamental resolution of Cerenkov radiation is determined by the distance over which a β-particle emits light. It was found that for ^90^Y and ^18^F, this distance is approximately 2 and 0.3 mm, respectively [22]. This shows that lower-energetic tracers have a better physical resolution limit, but the downside is a lower light yield, and thus, sensitivity.

## Characteristics of CLI from an image-guided surgery perspective

CLI images can be acquired by detecting the Cerenkov light from PET tracers using ultra-high-sensitivity optical cameras such as electron-multiplying charge-coupled device (EMCCD) cameras. The CLI image can be analysed semiquantitatively in photon radiance. CLI and PET are directly correlated due to both techniques measuring the photons produced by positron-emitting radiopharmaceuticals; PET measures the annihilation photons, and CLI measures the Cerenkov photons. Several studies have shown a strong correlation between CLI and PET for different radiopharmaceuticals in vitro, ex vivo and in vivo, thus demonstrating the feasibility of CLI for molecular imaging of living subjects. An overview of the published literature on the correlation between CLI and PET is provided in Table 2. Results on the correlation between CLI and radiotracer activity are also included in this table.

**Table 2:** Literature overview on the correlation of CLI and PET

CLI parameter	PET parameter	Correlation between CLI and PET	Radiopharmaceutical	In vivo, in vitro, ex vivo	Refs.
Radiance	%ID/g	*R* ^2^ = 0.93, 0.95, 0.93, 0.89	^18^F-FDG	In vivo	[71]
Radiance	%ID/g	*R* ^2^ = 0.97	^18^F-FDG	In vivo	[26]
Radiance	Activity	*R* ^2^ = 0.95	^18^F-FDG	In vivo	[72]
Radiance	Activity	*R* ^2^ = 0.98	^18^F-FDG	In vivo	[73]
Radiance	PET±	*P* = 0.02	^18^F-FDG	In vivo	[61]
Radiance	%ID/cm^3^	*R* ^2^ = 0.83	^18^F-FDG	In vivo	[74]
Radiance	%ID	*R* ^2^ = 0.82	^18^F-FDG	In vivo	[74]
Radiant vol.	Glycolytic vol.	*R* ^2^ = 0.99	^18^F-FDG	In vivo	[74]
Radiance	Activity	*R* ^2^ = 0.99	^18^F	In vitro	[75]
Radiance	Activity	*R* ^2^ = 0.97	^18^F-FDG	In vitro	[64]
Radiance	Activity conc.	*R* ^2^ = 0.99	^18^F-FDG	In vitro	[61]
Radiance	Activity	*R* ^2^ = 0.97	^18^F-FDG	Ex vivo	[72]
Intensity	Activity conc.	*R* ^2^ = 0.98	^68^Ga	In vitro	[76]
Intensity	Activity conc.	*R* ^2^ = 0.99	^68^Ga	In vivo	[76]
Radiance	%ID/g	R = 0.89	^89^Zr-trastuzumab	In vivo	[1]
Radiance	%ID/g	R = 0.98	^89^Zr-J591	In vitro	[28]
Radiance	Activity conc.	R = 0.98	^89^Zr-J591	In vitro	[28]
Radiance	%ID/g	*R* ^2^ = 0.85	^89^Zr-rituximab	In vivo	[77]
Radiance	Activity	*R* ^2^ = 0.98	Na-^131^I	In vitro	[68]
Radiance	Activity	*R* ^2^ = 0.99	^131^I-NGR	In vitro	[78]
Radiance	%IA/g	*R* ^2^ = 0.94, 0.98	^90^Y-DOTA-AR	In vivo	[79]
Radiance	%IA/g	*R* ^2^ = 0.91, 0.99	^90^Y-DOTA-AR	Ex vivo	[79]

There are several reasons why CLI has sparked so much interest in the field of biomedical imaging, and why it is a promising technology to guide surgical resection. Firstly, CLI images can be acquired with clinically approved tumour-targeted radiopharmaceuticals that have been used for over two decades in molecular medical imaging [26]. This provides great potential for rapid translation of CLI into clinical practice. Especially, the possibility to use the most commonly used PET radiopharmaceutical 2-deoxy-2-(^18^F)fluoro-d-glucose (abbreviated ^18^F-FDG) facilitates wide clinical adoption of CLI, as this is a versatile tracer that can be used in several solid cancers, including lung cancer, colorectal cancer, melanoma, head and neck cancer, breast cancer and oesophageal cancer [35].

The ability to use clinically approved tumour-specific tracers is an important advantage over conventional optical imaging techniques, such as targeted fluorescence imaging, as to date, there are no tumour-specific fluorescent tracers that have been approved by the FDA or EMA [36]. Targeted fluorescence imaging faces a significant commercial hurdle for clinical adoption, because the process of obtaining regulatory and reimbursement approval is costly and lengthy [37], while the revenue of imaging agents is often low compared to therapeutic agents, which makes it a significantly less interesting investment for industry [38, 39].

In addition to the already approved PET tracers, a significant number of new tracers are being developed for market approval including ^68^Ga-PSMA, ^68^Ga-DOTATOC, ^18^F-NaF, ^18^F-Choline, and ^18^F-FDOPA [40].

The ability to use the same tracer for both CLI and PET or SPECT enables dual-modality molecular imaging. PET and SPECT provide preoperative information on the location and extent of the tumour, while CLI can be used as an intraoperative adjunct to aid lesion identification and guide surgical resection. The use of the same tracer ensures visualisation of the same structures and facilitates a more accurate comparison between modalities. Depending on the patient pathway and half-life of the tracer, preoperative and intraoperative imaging could be performed using only one tracer injection, or by reinjecting the tracer. By capturing a white-light image with a standard camera at the time of CLI image acquisition, the functional information from the CLI image can be combined with the anatomical and structural information from the photograph, thereby providing the surgeon unprecedented information on the nature, location, and extent of the cancerous tissue.

Beta-emitting radiopharmaceuticals can also be detected by a beta probe or gamma probe [41–43], so these tools could potentially be used in addition to CLI-guided surgery to overcome the limited penetration depth of CLI as a result of absorption and scattering, thereby further ensuring successful tumour resection.

Another advantage of CLI is that the optical imaging systems required to acquire an image can be small in dimension or use fibre-optics or laparoscopic capabilities. Unlike a PET system, this provides the ability to use CLI in an operating theatre or in endoscopy equipment, and examples of such applications are provided in the next section.

CLI faces a number of challenges for routine clinical adoption. As mentioned earlier, Cerenkov luminescence is very faint due to the small number of optical Cerenkov photons emitted by charged particles. In biological applications, the signal intensity is further reduced by strong tissue attenuation from chromophores like (oxy)haemoglobin and light scattering which is more pronounced in the 400–650 nm range [44, 45]. Consequently, the acquisition time required to obtain high-resolution images with a sufficient signal-to-noise ratio (SNR) is longer than with conventional optical imaging. Typical imaging times in preclinical and clinical CLI studies range from 1 to 5 min (Table 3). Although these images are not available in ‘real-time’, these acquisition times are considered feasible for most intraoperative applications. However, when imaging with handheld devices (e.g. endoscopes), it is essential that during image acquisition, the device is not moved as this causes blurring of the image resulting in a reduced image quality. In an in vivo environment, this may prove especially difficult due to bowel activity and breathing artefacts, and motion-correction algorithms may be needed to correct for this.

**Table 3:** Overview of published studies on CLI-guided surgery

Preclinical or clinical	Indication	Tumour type	Tracer	Dose	CLI device	Acquisition time	Refs.
Preclinical	CLI-guided tumour resection	HER2+ breast cancer	^89^Zr-DFO-trastuzumab	4 MBq	Ivis optical imager	2–5 min	[1]
Preclinical	CLI-guided tumour resection	Glioblastoma	^68^Ga-3PRGD2	3.7 MBq	Ivis optical imager	1–5 min	[80]
Preclinical	Cerenkov luminescence endoscopy	Brain glioma	^18^F-FDG	37 MBq	Custom-build flexible fibre endoscope light-tight box	5 min	[62]
Preclinical	Cerenkov luminescence endoscopy	Glioblastoma	^90^Y-PRGD2, ^18^F-FP-PRGD2	8.1 MBq, 33 MBq	Custom-build flexible fibre endoscope light-tight box	6 min	[81]
Preclinical	Cerenkov luminescence endoscopy	Colon cancer	^18^F-FDG	24 MBq	Clinically approved rigid laparoscope coupled to EMCCD camera in light-tight box	5 min	[82]
Clinical	Cerenkov luminescence endoscopy	Rectal cancer	^18^F-FDG	9.25 MBq/kg	Clinically approved flexible fibre endoscope coupled to EMCCD camera	5 min	[73]
Preclinical	CLI-guided lymph node mapping	N/A	^68^Ga-SPIONs^a^	5–10 MBq	CCD camera positioned in light-tight box	2–10 min	[53]
Preclinical	CLI-guided lymph node mapping	N/A	^18^F-FDG	1.2 MBq	Ivis optical imager	2 min	[52]

^a^Superparamagnetic iron oxide particles (SPIONs)

The weak light intensity also requires a light-tight environment as any leakage of ambient light will overwhelm the CLI signal. Since Cerenkov radiation is strongest in the visible wavelengths, it cannot be spectrally separated from the much brighter ambient lights currently used in operating theatres. Control of the light environment is, therefore, currently achieved by imaging in a light-tight specimen chamber or room with light-sealed doors, or in anatomical areas that provide natural darkness (e.g. gastrointestinal tract).

An often mentioned limitation of optical imaging, in general, is the limited light penetration depth, and thereby, the inability to image deep located tissue. This was nicely illustrated by Chin et al. who calculated the reduction in signal intensity from one ^18^F-isotope and one ICG molecule in 1 mm of tissue, and found a reduction in signal intensity of 77 and 39 %, respectively [34]. Because Cerenkov light is ‘blue-weighted’ and tissue absorption and scattering are significantly increased for these wavelengths, CLI is mainly applicable for imaging superficially located tissue.

Due to the half-life dependency of radiotracers, the window in which CLI imaging needs to be performed to obtain a sufficient SNR and image quality is limited. Well-designed logistics and close collaboration between nuclear medicine, radiology and surgical departments are, therefore, a prerequisite for the successful implementation of CLI in current clinical and surgical workstreams.

A challenge for CLI-guided surgery in particular is the radiation exposure to patients and theatre staff from using radiopharmaceuticals. For patients, the effective dose from a 300 MBq ^18^F-FDG injection is approximately 6 mSv; this is comparable to the radiation dose for a typical chest CT scan [46] and much lower than the 20–2500 mSv radiation exposure from diagnostic and interventional fluoroscopy procedures [47]. Staff that work in close proximity of the patient during surgery are also exposed to radiation. The received radiation dose is dependent on the time between injection and the start of the interventional procedure, as well as the duration of the procedure. Various groups have published staff radiation doses from ^18^F-FDG-guided cancer surgery procedures [48–50], and have shown that the radiation dose received per procedure is generally low. For example, for a 105 min procedure starting approximately 1 h after injection of 370 MBq ^18^F-FDG, the exposure to the surgeon was 42 μSv [48]. However, depending on the national annual occupational dose limit (50 mSv in the United States, and 20 mSv in most other countries) and type of procedure, the number of procedures an individual can perform per year without exceeding the permissible limits for professional workers may be restricted. Regardless of these limits, there are strict requirements for the use of radioactivity in clinical practice. For example, routine staff monitoring is a requisite for each institution that conducts radiotracer guided procedures, strict regulations need to be followed with regards to clinical waste disposal and handling of radioactive specimens, and staff need to attend radiation safety training prior to participation in any procedure involving radiation [51]. These requirements could hinder adoption of radioguided surgical technologies, especially in small district hospitals that do not have access to nuclear medicine or radiation safety departments. The aforementioned characteristics of Cerenkov radiation and CLI in light of image-guided surgical applications are summarised in Table 1.

## Applications of CLI for image-guided surgery and ongoing clinical trials

After it was first described in 2009, CLI has gained significant scientific interest. A search of Embase and Medline performed on 28 December 2015 using the keywords ‘Cerenkov Luminescence Imaging’ provided a total of 103 and 59 articles, respectively. Despite the limitations mentioned in the previous section, various research groups have been successful in using CLI for image-guided surgical interventions. An overview of the results published to date is provided in Table 3. The majority of this work is preclinical, although one clinical study was also published recently. In addition to the tumour types shown in Table 3, CLI-guided surgery could also be applied to other superficial malignancies where precision surgery is essential for preserving organ function, such as neoplasms in the oral cavity and genital tract. However, publications of CLI in these malignancies have not yet emerged.

**Fig. 2:**
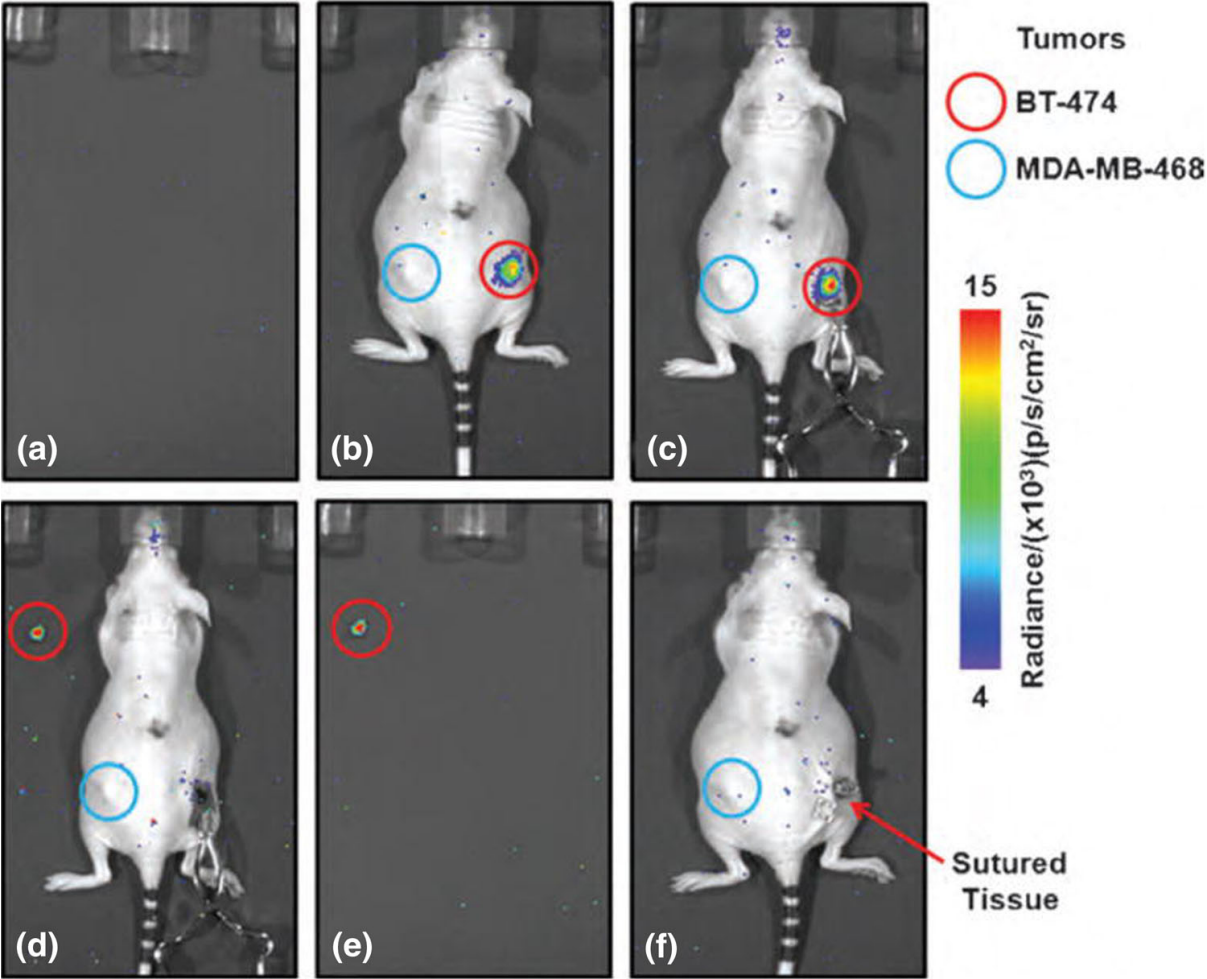
^89^Zr-DFO-trastuzumab CLI-guided tumour excision. **a** Empty background image acquired prior to surgery. **b** Image acquired pre-incision and **c** post-incision after removal of the skin. An elevated tumour radiance is visible in the HER2/neu positive tumour (*red circle*); ^89^Zr-DFO-trastuzumab is not taken up in the HER2/neu negative tumour, and this tumour, therefore, does not display an elevated radiance (*blue circle*). Note the increase in radiance due to a reduction in tissue absorption and scattering after removal of the skin. **d** Image of the surgical cavity after excision of the HER2/neu positive tumour. An elevated radiance from the excised tumour specimen is visible (*red circle*). No CLI signal is left at the excision site indicating complete tumour resection. **e** Image of excised tumour alone. **f** Image acquired straight after the surgical wound was closed with sutures. This research was originally published in Molecular Imaging [1]

The published studies show the ability to perform CLI-guided surgical excision of tumours using a variety of radiopharmaceuticals and different CLI embodiments, including standard IVIS optical imaging systems, custom-build flexible fibre endoscope systems, and clinically approved rigid laparoscope and flexible endoscope systems coupled to EMCCD cameras. An example that nicely illustrates CLI-guided tumour excision is shown in Fig. 2.

An important advantage of using CLI in an endoscopic setting is that these make use of anatomical dark chambers, so that there is no interference from external light sources. Besides, this technology can also be implemented in other types of endoscopes, such as a bronchoscope or hysteroscope, and future applications of CLI could, for example, focus on lung cancer, endometrial cancer and metastatic lymph nodes in the abdomen, pelvis and thorax.

CLI has also been successfully used for lymph node identification and image-guided lymph node excision using ^18^F-FDG and ^68^Ga-labelled superparamagnetic iron oxide particles (SPIONs) [52, 53].

Another interesting application of CLI, although not directly related to image-guided surgery, has been published by Spinelli et al. [54]. They imaged the thyroid gland of a patient treated for hyperthyroidism who received 550 MBq of Iodine-131 (^131^I). Using an EMCCD camera positioned in a light-tight room, tracer uptake in the thyroid could be visualised with a 2-min exposure time. This application is of clinical interest as imaging the uptake of beta-emitting radiopharmaceuticals could provide a rapid and inexpensive alternative for monitoring radiation doses given to superficial organs.

**Fig. 3:**
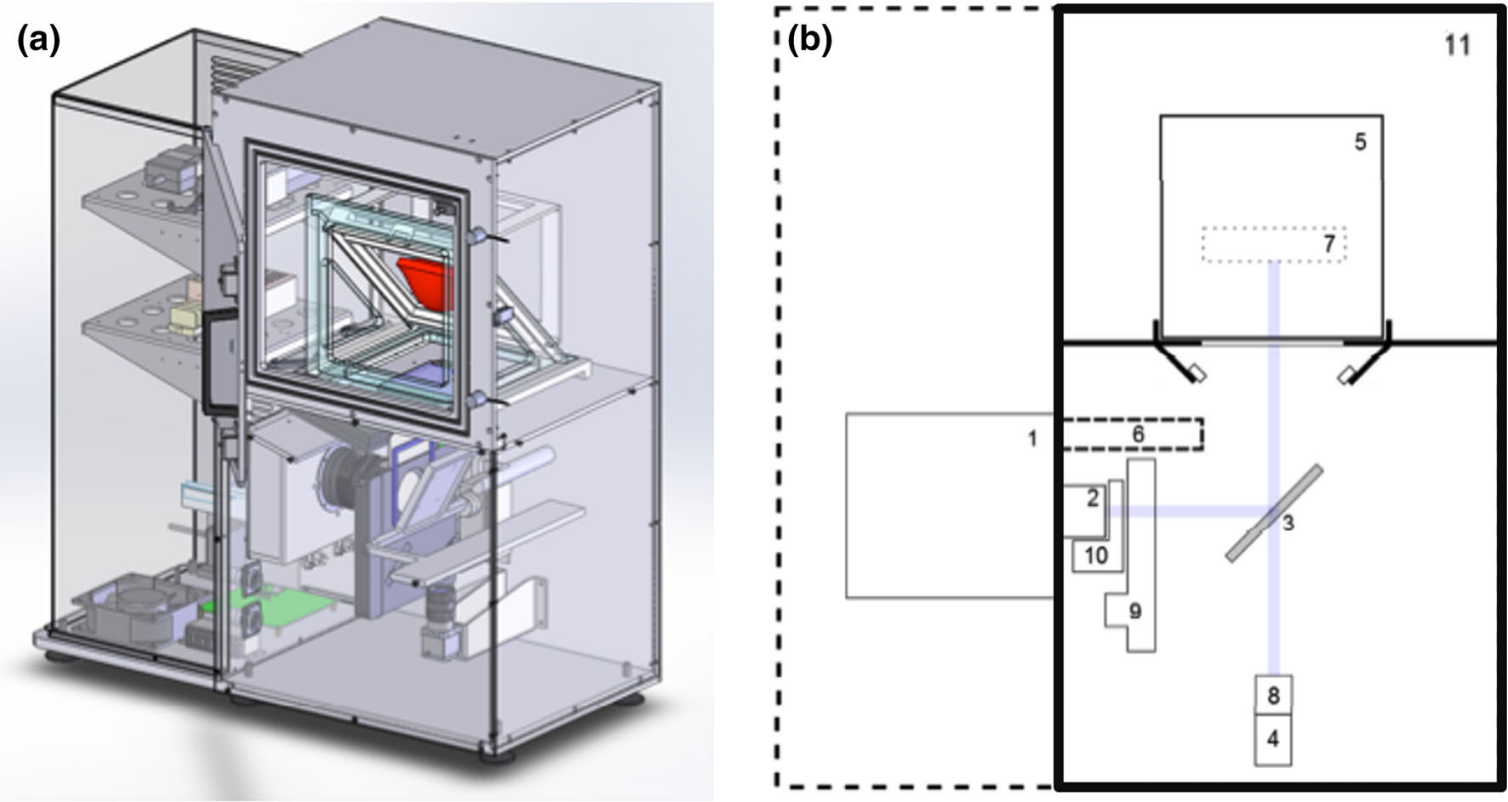
Investigational intraoperative CLI imaging system used in breast-conserving surgery trial. **a** Computer aided design (CAD) rendering. The *red* object indicates the location of the tissue specimen within the specimen chamber. **b** Schematic diagram showing: (*1*) thermoelectrically-cooled EMCCD camera, (*2*) *f*/0.95 lens, (*3*) hinged reflex mirror, (*4*) CMOS reference camera for anatomical imaging, (*5*) specimen holder, (*6*) lead radiation shielding for EMCCD camera, (*7*) focal zone, (*8*) fixed lens for reference camera, (*9*) filter wheel, (*10*) LED RGB light array, (*11*) specimen chamber. The *purple line* shows the optical paths for the EMCCD camera and the reference camera as determined by the angle of the reflex mirror

**Fig. 4:**
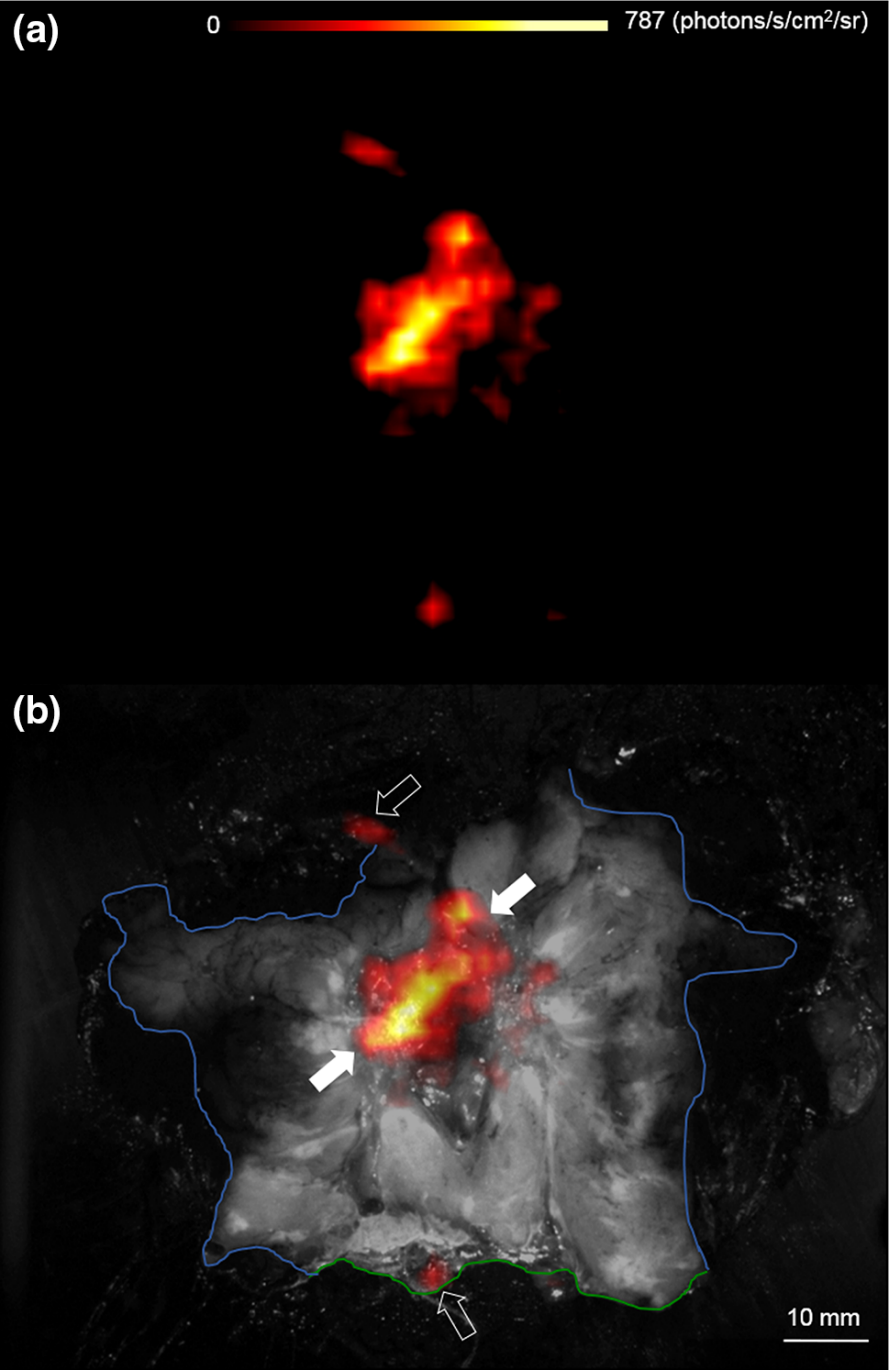
Wide local excision specimen from a patient with a 22 mm, grade 2, ER+/HER2− invasive lobular carcinoma. The specimen was incised to expose the primary tumour and margins of excision, and subsequently scanned with the investigational CLI camera. **a** Cerenkov image, **b** white-light photograph (*black* and *white*) overlaid with Cerenkov signal. An increased radiance from the tumour is visible (*white arrows*); mean radiance is 544.0 (SD 71.0) photons/s/cm^2^/sr. The tumour-to-tissue background ratio is 2.44. Phosphorescent signals from the pathology inks used to orientate the specimen prior to incision are also present (*open arrows*). The posterior margin (*blue*) and superior margin (*green*) are visible; both margins were clear (≥5 mm) on CLI and histopathology, respectively

The successful applications of CLI for image-guided cancer surgery have resulted in several clinical studies that are currently ongoing to evaluate the feasibility of this technique in different tumour types. At Guy’s Hospital (London, UK), a first-in-woman pilot study evaluates intraoperative CLI for measuring tumour resection margins and lymph node status in 30 patients undergoing breast-conserving surgery (BCS) (ClinicalTrials.gov identifier NCT02037269). Patients receive an intravenous standard of care PET dose of 5 MBq/kg ^18^F-FDG, and excised wide local excision (WLE) specimens and lymph nodes are imaged within 1–3 h post-injection using an investigational intraoperative CLI specimen camera (Lightpoint Medical Ltd, UK) (Fig. 3). The investigational CLI camera consists of a light-tight sample chamber, a radiation-shielded thermoelectrically-cooled EMCCD camera, and a *f*/0.95 lens. The camera provides 8 × 8 cm field of view and 156 µm intrinsic spatial resolution. Interim results show that elevated radiances are detected in cancer compared to normal breast tissue, and that the radiation exposure to surgical staff is low [55, 56]. The results from comparing CLI resection margin status and lymph node status to the gold-standard, histopathology, are being prepared for publication at the time of writing. An example of a CLI image from a WLE specimen that was scanned intraoperatively in this clinical study is shown in Fig. 4. This image illustrates that CLI provides high-resolution functional information that allows surgeons to accurately assess tumour margins during surgery.

To evaluate the effect of intraoperative 18F-FDG CLI on reoperation rate and quality of life in BCS, a randomised, controlled, multi-centre clinical study is scheduled to commence in mid-2016 (ClinicalTrials.gov identifier NCT02666079). This will run across an anticipated eight study sites in the UK and Germany, and use the CE-marked LightPath™ Imaging System (Lightpoint Medical Ltd, UK).

Another CLI study that is currently being conducted at Guy’s Hospital and University College London Hospital focusses on tumour margin evaluation in prostate cancer (ClinicalTrials.gov identifier NCT02151097). Patients undergoing a therapeutic radical prostatectomy receive a 370 MBq intravenous injection of ^18^F-Choline, and the margins of the resected prostatectomy specimen are imaged using the investigational intraoperative CLI camera. The initial results show that intraoperative ^18^F-Choline CLI is a feasible and low-risk procedure [57]. Elevated radiances were present in all three primary tumours (tumour-to-background ratio between 2.49 and 4.90), and CLI imaging did not add additional time to surgery. The assisting surgeon and scrub nurse received the highest body dose; 110–180 and 40–80 μSv, respectively. Work is currently being done to perform CLI imaging with the Gallium-68 (^68^Ga) labelled prostate specific membrane antigen (PSMA); a tracer that has strong advantages over ^18^F-Choline. PSMA is a cell surface target that is highly expressed by nearly all prostate cancers, and ^68^Ga-PSMA is, therefore, highly taken up in prostate cancer cells [58]. The Cerenkov radiance from ^68^Ga in tissue (*η* = 1.4) is approximately 17 times higher compared to ^18^F, which could facilitate a reduction in tracer dose, thereby lowering the radiation exposure to theatre staff. The shorter ^68^Ga half-life of 68 min means that contaminated surgical instruments and surgical waste can be cleaned and disposed much quicker. Another advantage is that besides imaging the primary tumour, this tracer also holds promise for visualising small lymph node metastases [59].

In addition to imaging resected WLE specimens ex vivo, scanning the post-resection surgical cavity for residual tumour that cannot be identified by visual inspection or palpation could further aid achieving complete excision of cancers. Detection of beta-radiation with handheld betascopes can identify small areas of malignant cells [60], and clinical studies to test the combination of in vivo betascope detection and ex vivo CLI will soon commence in gastrointestinal cancers (ClinicalTrials.gov identifier NCT02446379) and breast cancer (ClinicalTrials.gov identifier NCT02151071).

Another interesting application of CLI that is currently being evaluated is the non-invasive detection of nodal disease in a preoperative clinical setting (ClinicalTrials.gov Identifier NCT01664936). In this observational study, patients with lymphoma, leukaemia and metastatic lymphadenopathy scheduled to undergo standard clinical ^18^F-FDG PET are included. CLI imaging is performed immediately after the PET-scan in a dark room with a single-photon sensitive camera positioned on a standard photography tripod. The preliminary results of this study from four patients (two lymphoma, one lung cancer and one breast cancer) showed that metastatic lymph nodes in the neck or axilla, located at 1.6 ± 0.5 cm under the skin, had a statistically significant higher Cerenkov signal than negative nodes (*P* = 0.02), and this finding strongly correlated with the results from PET [61]. Examples of patient population that can benefit from accurate preoperative identification of nodal disease are breast cancer patients with involved lymph nodes. If positive lymph nodes are identified preoperatively on CLI, their treatment could convert from sentinel lymph node biopsy (SLNB) to immediate axillary node clearance (ANC), thus preventing the patient from undergoing an unnecessary surgical procedure. Alternatively, these patients may undergo neoadjuvant chemotherapy followed by SLNB ± ANC. Completion of this study will provide further insight in the real value of preoperative CLI imaging in aiding surgical and medical decision-making.

## Future technical developments

CLI has only recently been introduced as a modality for imaging biological tissue, and this technique is, therefore, still in its infancy. In the last decade, advances in optical imaging devices in the biophotonics field have progressed rapidly with the development of highly sensitive, charge-coupled detectors (CCD), and current technological developments focus on further increasing the sensitivity of this imaging technology. This would facilitate a reduction in acquisition time, and a reduction in the administered radiopharmaceutical dose.

Improvements in detection sensitivity can be achieved using more specialised optics and more sensitive detectors. For example, the Schott-75 glass used in the CLI prototype device of Liu et al. transmits only 40 % of light at 500 nm, and impurities in the glass scintillate gamma photons, which increase background noise [62]. The use of fused silica, which transmits further in the violet and ultraviolet wavelengths and has fewer impurities, would significantly improve detection sensitivity.

In non-invasive CLI imaging, improvements in sensitivity may be obtained by using CCD cameras that are optimised to detect Cerenkov radiation in the UV for surface imaging, or in the near-infrared (NIR) for deep imaging. Spinelli et al. showed in a theoretical analysis that a CCD detector with a quantum efficiency peak in the NIR range could enhance the number of detected Cerenkov photons by 35 %, especially for Cerenkov source located deeper inside the tissue [63].

As already described in the section ‘Cerenkov radiation’, Cerenkov light is mainly weighted towards the ultraviolet (UV) and blue part of the spectrum. The high absorption and scattering of these frequencies in biological tissue hampers CLI detection and quantification. To overcome these limitations, current work focusses on shifting the CLI emission spectrum to NIR wavelengths by ways of Cerenkov radiation energy transfer (CRET). Different research groups have done this using fluorescent quantum dots (QDs) or other fluorophores, in vitro and in vivo animal models [64–66]. The broad excitation spectrum that matches the CR spectrum and the narrow emission spectrum make QDs specifically favourable. NIR wavelength light would enable the use of spectral filters to reduce interference from external light source, thus facilitating the use of CLI in the intraoperative suite. However, as with targeted fluorescent probes, nanoparticles have not yet received marked approval, and these approaches can, therefore, not be used clinically yet.

Another interesting development in the field of CLI is the acquisition of three-dimensional (3D) images by means of Cerenkov luminescence tomography (CLT). Different reconstruction approaches have been proposed using multi-view [67, 68] or multi-spectral [69] imaging methods, all showing a good correlation in radiotracer distribution on CLT and PET or SPECT, respectively. Although each method is currently still limited in terms of acquisition time or spatial resolution and has only been used preclinically, the ability of CLT to provide 3D information on the in vivo distribution of radiopharmaceuticals could provide a more accurate depiction of the location and extent of the tumour, thereby aiding the surgeon in more accurate tumour excision.

## Conclusions

CLI is a fast-emerging optical imaging technology that has rapidly progressed from bench to bedside. This rapid development has been facilitated by the ability to use clinically approved tumour-targeted PET tracers. Due to its high-resolution, wide applicability across a range of solid cancers and small size imaging equipment, CLI is of particular interest in the field of image-guided surgery. Challenges for the clinical implementation of this technique include the low signal intensity, the requirement for light-tightness, the minute-scale image acquisition times and the logistical issues associated with using radiotracers intraoperatively. Preclinical studies have shown that CLI can be successfully used to guide surgical resection of tumours and lymph nodes, as well as to detect cancerous lesions using Cerenkov luminescence endoscopy. Several clinical studies on the preoperative and intraoperative use of CLI in breast cancer, prostate cancer, gastrointestinal cancer and metastatic lymph nodes are currently underway. Results from these studies, together with ongoing developments in ultra-sensitive camera technology will help drive widespread clinical adoption. By improving the accuracy of surgical resections, CLI has the potential to become a disruptive technology in cancer surgery.
